# Role of Hybrid Fixator in Proximal Tibial Fractures: A Prospective Evaluation of Clinical and Radiological Outcomes

**DOI:** 10.7759/cureus.109850

**Published:** 2026-05-28

**Authors:** Happy Kumar, Jagdeep S Rehncy, Girish Sahni, Arvind Kumar, Harsh Kumar, Devashish Gautam

**Affiliations:** 1 Department of Orthopaedic Surgery, Government Medical College Patiala, Patiala, IND; 2 Department of Orthopaedics, Government Medical College Patiala, Patiala, IND; 3 Department of Orthopaedics and Traumatology, Government Medical College, Patiala, Patiala, IND; 4 Department of Orthopaedics and Traumatology, Rajindra Hospital, Patiala, IND

**Keywords:** external fixation (umex), fracture healing factors, major trauma, proximal tibial fractures, road traffic injuries

## Abstract

Background

Management of complex proximal tibial fractures is challenging due to periarticular comminution and associated soft-tissue compromise. Hybrid external fixation has been proposed as an alternative to internal fixation in such cases. This study aimed to evaluate the functional and radiological outcomes of hybrid external fixation in proximal tibial fractures.

Methods

This was a prospective observational study conducted at a tertiary care teaching hospital over one year. Thirty adult patients with periarticular or intra-articular proximal tibial fractures were treated using hybrid external fixation. Patients were followed up for a mean duration of six months. Functional and radiological outcomes were assessed at final follow-up using Rasmussen’s functional and radiological scoring systems. Descriptive statistical analysis was performed.

Results

Thirty patients with complex proximal tibial fractures were included. The mean age was 48.2 ± 11.86 years, with a male predominance (63.33%). Road traffic accidents accounted for 83.33% of injuries, and most fractures were Schatzker type V (36.67%) or VI (43.33%). The mean injury-to-surgery interval was 2.7 ± 1.1 days, and postoperative weight bearing was initiated at 8.68 ± 1.26 weeks. The mean Rasmussen functional score was 26.37 ± 3.3, with excellent-to-good functional outcomes in 83.34% of patients. Excellent-to-good radiological outcomes were achieved in 86.67%. Pin-tract infection was the most common complication (16.67%), while 63.33% of patients had no complications.

Conclusion

Hybrid external fixation resulted in acceptable short-term functional and radiological outcomes in complex proximal tibial fractures, particularly following high-energy trauma with soft-tissue compromise. The procedure demonstrated a manageable complication profile; however, conclusions are limited by the small sample size, descriptive design, and short follow-up. Larger comparative studies are required to better define its role in this fracture subset.

## Introduction

Proximal tibial fractures constitute a complex group of periarticular injuries that frequently result from high-energy mechanisms such as road traffic accidents and falls from height [1]. These fractures are often associated with articular comminution, metaphyseal instability, and varying degrees of soft-tissue compromise, all of which complicate fracture management and rehabilitation [2]. Achieving stable fixation while preserving the soft-tissue envelope and restoring knee function remains a key challenge in the treatment of these injuries [1,2].

Multiple treatment modalities have been described for proximal tibial fractures, including conservative management, open reduction and internal fixation (ORIF), circular external fixation, and hybrid external fixation systems [3]. ORIF allows direct visualization and anatomical reduction of the articular surface but frequently necessitates extensive soft-tissue dissection, which may increase the risk of wound complications, infection, and delayed healing, particularly in high-energy fractures with compromised soft tissues [4]. These concerns are especially relevant in clinical settings where delayed presentation, open injuries, and limited access to advanced soft-tissue reconstruction are common [5].

Hybrid external fixation has emerged as an alternative strategy that combines circular fixation in the periarticular region with uniplanar fixation of the diaphyseal segment [6]. This construct aims to provide adequate fracture stability while minimizing additional soft-tissue trauma and avoiding knee-spanning immobilization, thereby facilitating early mobilization. Several studies have reported acceptable outcomes with hybrid external fixation in complex proximal tibial fractures [6]. However, much of the existing evidence is derived from retrospective analyses, heterogeneous cohorts, or studies conducted in high-resource environments, limiting the generalizability of their findings.

Notably, there remains a relative paucity of prospective data evaluating functional and radiological outcomes of hybrid external fixation using standardized assessment tools, particularly from Indian and resource-constrained settings [7]. Differences in injury patterns, soft-tissue status, rehabilitation protocols, and healthcare infrastructure may significantly influence outcomes, underscoring the need for context-specific evidence. Prospective evaluation in such settings is essential to better understand the feasibility, short-term outcomes, and complication profile of hybrid external fixation in routine clinical practice [7].

This prospective study evaluates short-term functional and radiological outcomes of hybrid external fixation in complex proximal tibial fractures in a tertiary care setting.

The present study was undertaken to evaluate the functional and radiological outcomes of hybrid external fixation in the management of complex proximal tibial fractures. Additionally, the study aimed to assess the time to fracture union, determine the incidence and nature of complications, and analyze factors influencing functional outcomes in these patients.

## Materials and methods

Study design and setting

This study was designed as an exploratory, prospective, observational, non-comparative investigation conducted in the Department of Orthopaedics at Government Medical College and Rajindra Hospital, Patiala, India, a tertiary care teaching hospital. The study was carried out over a one-year period from December 2024 to December 2025 (approval no. BFUHS2k26p-TH280).

Study population and sample size

Adult patients admitted with proximal tibial fractures were consecutively screened for eligibility according to predefined inclusion and exclusion criteria. A total of 30 patients were enrolled during the study period. The sample size was determined pragmatically, reflecting the number of eligible patients presenting during the study duration. Given the exploratory and non-comparative nature of the study, a formal sample size calculation was not performed.

Eligibility criteria

Patients aged 18 years or older, of either sex, with periarticular or intra-articular proximal tibial fractures were included. This comprised closed tibial plateau fractures classified as Schatzker type V and VI, as well as open periarticular or intra-articular compound fractures in which primary internal fixation was considered unsuitable due to soft-tissue compromise. Patients younger than 18 years, those unwilling to provide informed consent, individuals with associated neurovascular injuries, and patients deemed medically unfit for surgery were excluded.

Intervention

All enrolled patients were treated using a hybrid external fixation technique, consisting of a circular ring construct applied to the periarticular region, combined with a uniplanar external fixator for diaphyseal stabilization. Standard trauma assessment and stabilization protocols were followed prior to surgical intervention in all cases.

Outcome measures

The primary outcome measures were functional and radiological outcomes, assessed using Rasmussen’s functional and radiological scoring systems at final follow-up [8]. The functional score evaluates pain, walking capacity, range of motion, stability, and extension lag, while the radiological score assesses articular depression, condylar widening, limb alignment, and joint congruity.

Follow-up protocol

Patients were followed at regular postoperative intervals according to institutional protocol. The mean follow-up duration was 6.0 ± 1.4 months (range: 4-9 months). Functional and radiological assessments were performed at the final follow-up visit.

Statistical analysis

Data were recorded and analyzed using IBM SPSS Statistics for Windows, Version 26 (Released 2018; IBM Corp., Armonk, NY, USA). Statistical analysis was descriptive in nature. Continuous variables were summarized as mean ± standard deviation, and categorical variables were expressed as frequencies and percentages. No hypothesis testing or inferential statistical analysis was performed, in keeping with the exploratory design of the study.

## Results

A total of 30 patients with complex proximal tibial fractures treated using hybrid external fixation were included in the study.

Demographic and injury characteristics

**Figure 1:**
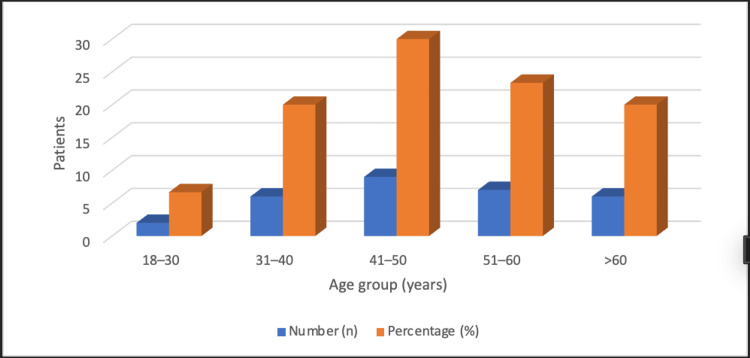
Age-wise distribution of patients (n = 30) The X-axis represents age groups (years), and the Y-axis represents the number of patients.

**Figure 2:**
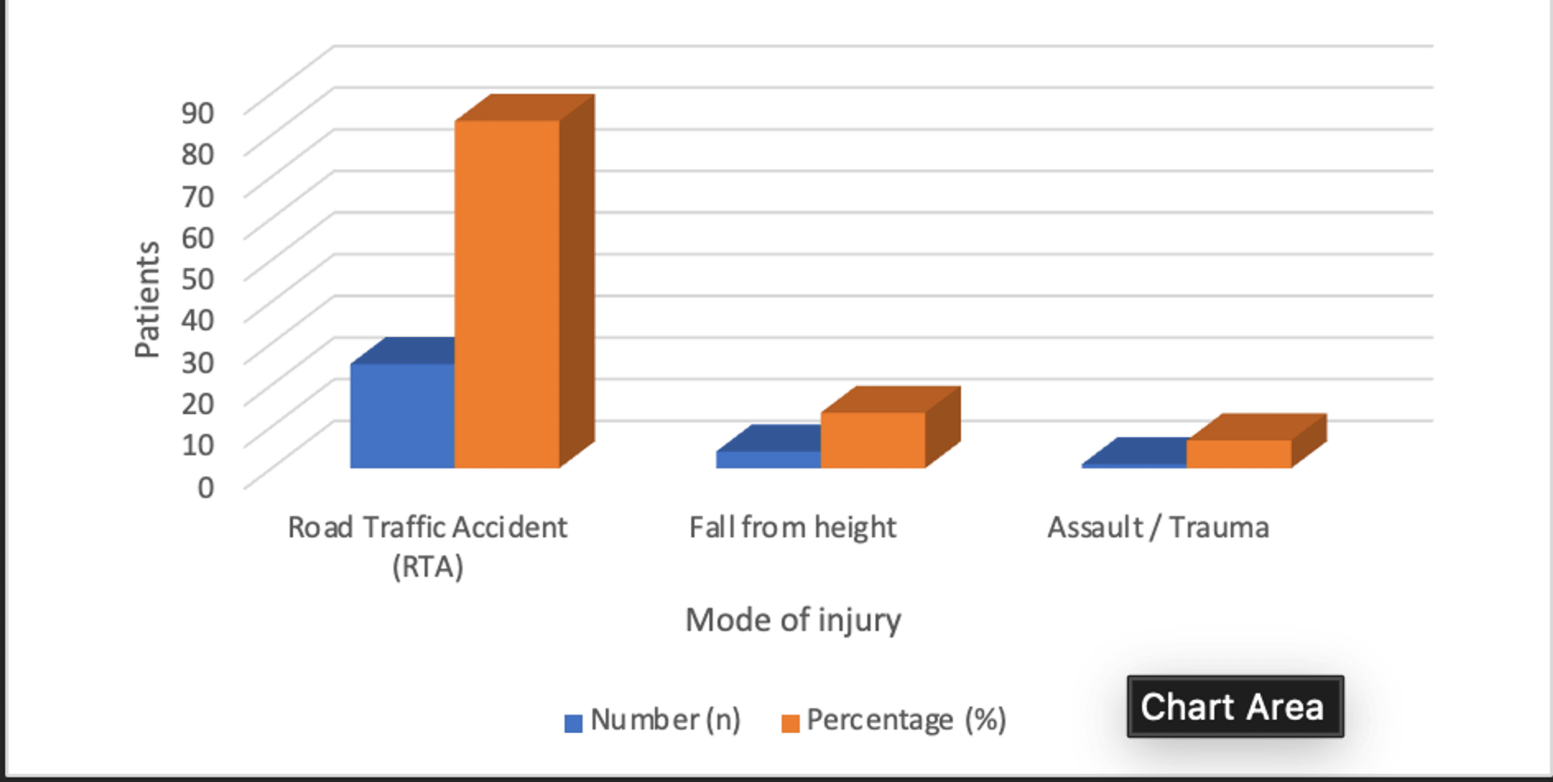
Mode of injury (n = 30) The X-axis represents the mechanism of injury, and the Y-axis represents the number of patients.

The mean age of the study population was 48.2 ± 11.86 years, with a male predominance of 63.33% (n = 19). Most patients were between 41 and 60 years of age (53.33%, n = 16). The right side was involved in 56.67% (n = 17) of cases. Road traffic accidents were the predominant mechanism of injury (83.33%, n = 25). Fracture pattern analysis showed that the majority were high-energy injuries, including Schatzker type VI (43.33%, n = 13) and type V (36.67%, n = 11) fractures, while open periarticular/intra-articular fractures accounted for 20% (n = 6) (Tables 1-2 and Figures 1-2).

**Table 1:** Fracture type (n = 30)

Fracture Type	Number (n)	Percentage (%)
Closed Schatzker V	11	36.67
Closed Schatzker VI	13	43.33
Open periarticular/intraarticular compound fracture	6	20
Total	30	100

**Table 2:** Gender-wise distribution (n = 30)

Gender	Number (n)	Percentage (%)
Male	19	63.33
Female	11	36.67
Total	30	100

Perioperative characteristics

The mean interval between injury and surgery was 2.7 ± 1.1 days. The mean duration of surgery was 93.1 ± 14.18 minutes, and the mean hospital stay was 7.4 ± 1.3 days. Postoperative weight bearing was initiated at a mean of 8.68 ± 1.26 weeks. Detailed perioperative distributions are provided in Appendix 1.

Radiological outcomes

**Figure 3:**
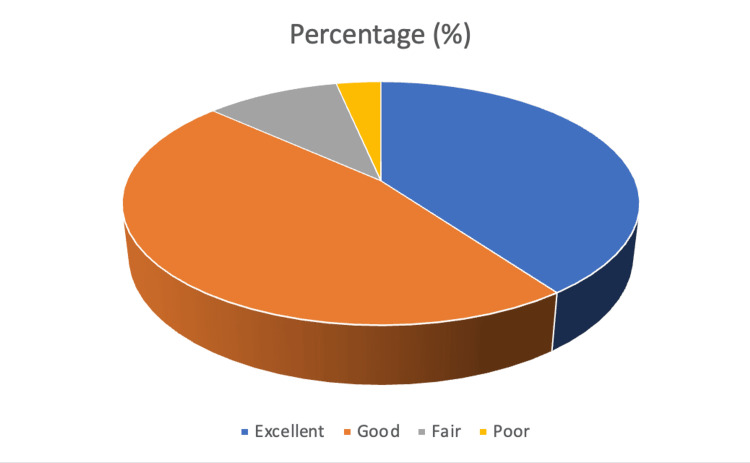
Radiological outcome (Rasmussen’s radiological score) Excellent outcomes were achieved in 12 patients (40%), and good outcomes in 14 patients (46.67%). Only 3 patients (10%) had fair outcomes, while 1 patient (3.33%) had a poor outcome. Therefore, excellent-to-good radiological alignment and healing were obtained in the majority (86.67%) of patients. The X-axis represents outcome categories (excellent, good, fair, poor), and the Y-axis represents the percentage of patients.

Radiological assessment using Rasmussen’s radiological scoring system demonstrated excellent outcomes in 40% (n = 12) and good outcomes in 46.67% (n = 14) of patients, fair outcomes were observed in 10% (n = 3), while poor outcomes were seen in 3.33% (n = 1) [8]. Overall, excellent-to-good radiological outcomes were achieved in 86.67% (n = 26) of cases (Figure 3).

Functional outcomes

**Figure 4:**
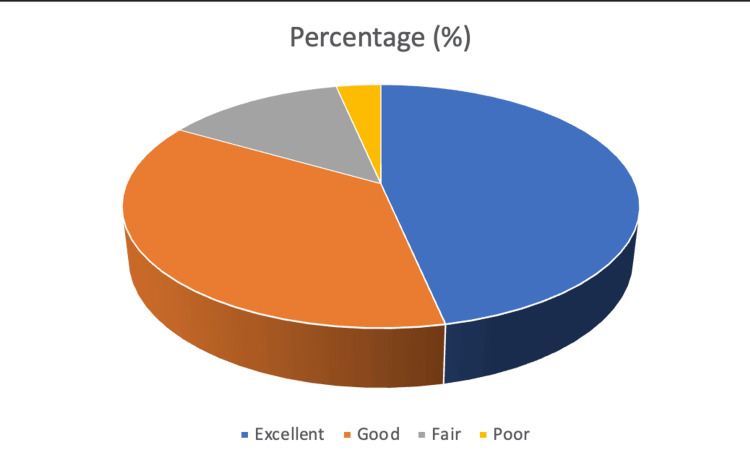
Clinical outcome (Rasmussen’s clinical score) Excellent clinical results were observed in 14 cases (46.67%), and good results in 11 cases (36.67%). Fair results were seen in 4 patients (13.33%), while a poor outcome occurred in 1 patient (3.33%). Hence, excellent-to-good clinical recovery was observed in 83.34% of patients, indicating satisfactory restoration of knee function and stability. The X-axis represents outcome categories (excellent, good, fair, poor), and the Y-axis represents the percentage of patients.

At final follow-up, the mean Rasmussen functional score was 26.37 ± 3.3. Excellent functional outcomes were observed in 46.67% (n = 14), and good outcomes in 36.67% (n = 11) of patients. Fair outcomes were seen in 13.33% (n = 4), while poor outcomes occurred in 3.33% (n = 1). Thus, excellent-to-good functional outcomes were achieved in 83.34% (n = 25) of patients (Table 3 and Figure 4).

**Table 3:** Rasmussen’s functional score at final follow-up

Rasmussen’s functional score	Number
Mean	26.37
SD	3.3

Postoperative complication

Pin-tract infection was the most common complication, occurring in 16.67% (n = 5) of patients. Joint stiffness and malunion were each observed in 6.67% (n = 2), while secondary infection also occurred in 6.67% (n = 2). Non-union and knee instability were each noted in 3.33% (n = 1). Notably, 63.33% (n = 19) of patients had no complications (Table 4).

**Table 4:** Complications observed (n = 30) The most common complication was pin-tract infection, noted in 5 patients (16.67%), which is a known complication of external fixation. Joint stiffness and malunion were each observed in 2 patients (6.67%), while secondary infection occurred in 2 cases (6.67%). Non-union and knee instability were each noted in 1 patient (3.33%). Importantly, 19 patients (63.33%) had no complications, reflecting an overall acceptable safety profile and favourable outcomes with hybrid fixator application.

Complication	Number (n)	Percentage (%)
Pin tract infection	5	16.67
Joint stiffness	2	6.67
Malunion	2	6.67
Non-union	1	3.33
Secondary infection	2	6.67
Knee instability	1	3.33
No complication	19	63.33

**Figure 5:**
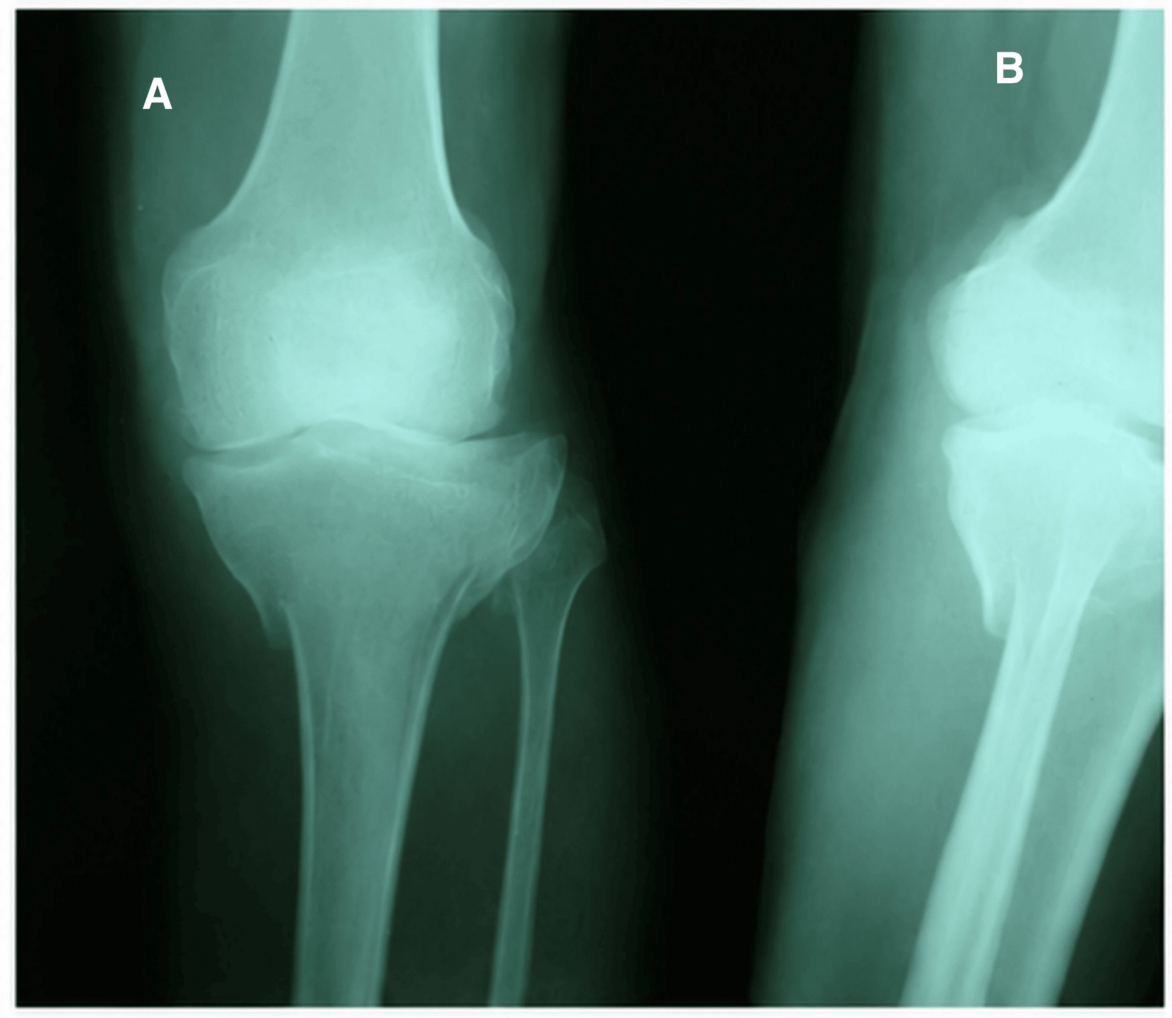
Preoperative anteroposterior (A) and lateral (B) radiographs demonstrating the intra-articular fracture pattern

**Figure 6:**
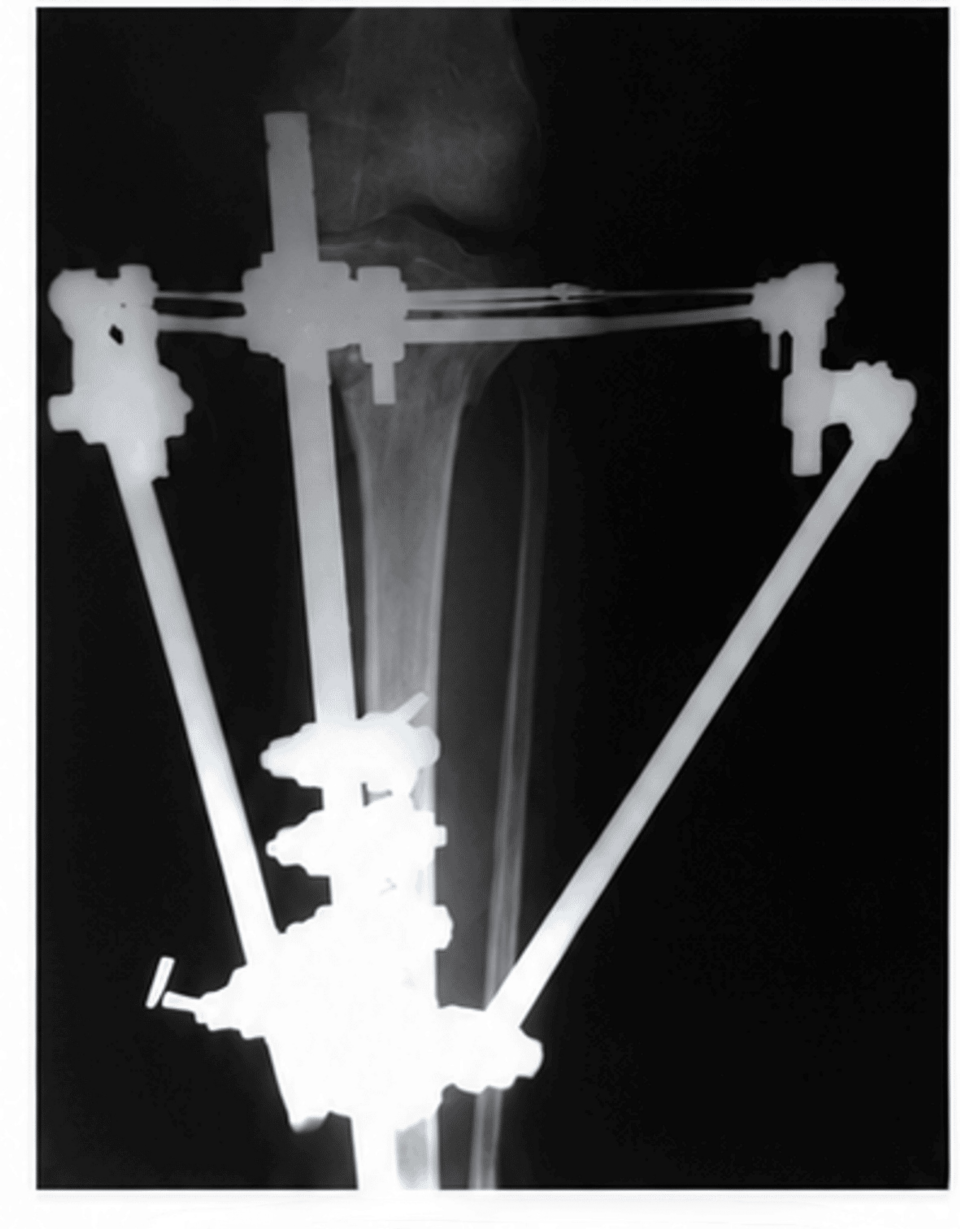
Postoperative radiographic image of a patient with hybrid external fixator fixation

**Figure 7:**
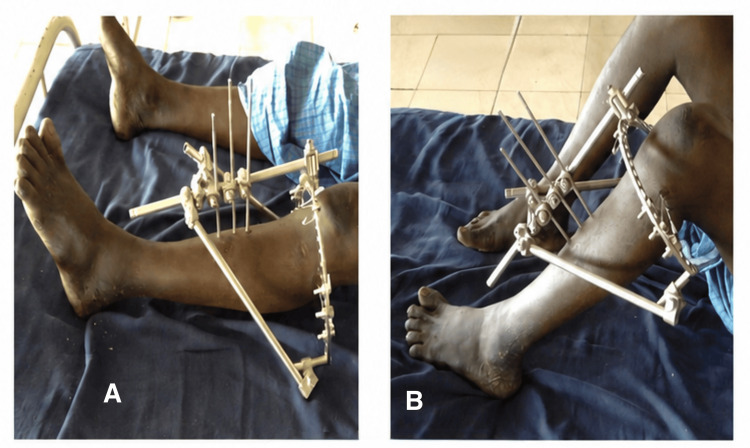
Three-month postoperative functional range of motion of the patient fixed with a hybrid fixator, in extension (A) and flexion (B)

**Figure 8:**
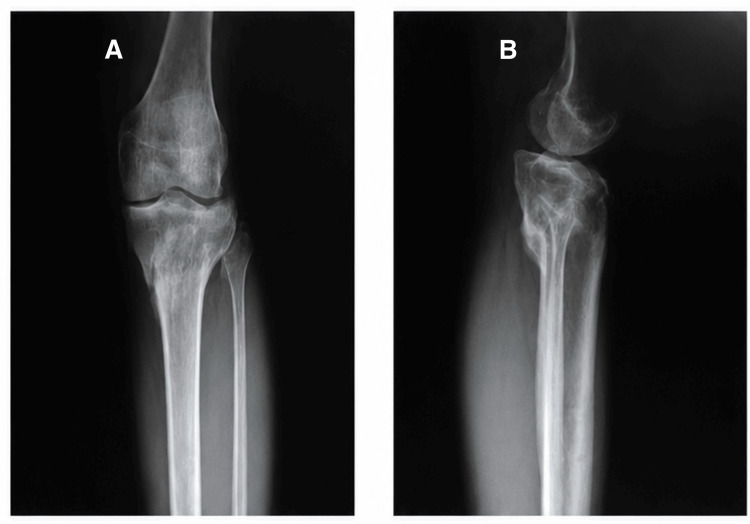
Four-month postoperative anteroposterior (A) and lateral (B) radiographs demonstrating satisfactory coronal alignment and bridging callus

A 35-year-old male patient presented with a history of a road traffic accident resulting in an injury to the knee region. Clinical examination revealed a closed proximal tibial injury without neurovascular deficit. Radiological evaluation demonstrated a Schatzker type V tibial plateau fracture (Figure 5). The patient was managed with hybrid external fixation (Figure 6). Postoperative recovery was uneventful, and standard rehabilitation protocols with gradual mobilization were followed. At follow-up of three months with the hybrid fixator in situ, the patient demonstrated a good functional outcome with satisfactory knee stability (Figure 7). The external fixator was removed at four months postoperatively following satisfactory radiological union (Figure 8).

## Discussion

This prospective observational study evaluated short-term functional and radiological outcomes of hybrid external fixation in complex proximal tibial fractures. The study cohort predominantly sustained high-energy injuries, with road traffic accidents accounting for 83.33% of cases, and most fractures classified as Schatzker type V and VI, including open periarticular injuries. Such fracture patterns are frequently associated with significant soft-tissue compromise, making stable fixation and early rehabilitation challenging.​​​​

The demographic profile showed a predominance of middle-aged males (mean age 48.2 ± 11.86 years), consistent with the epidemiology of high-energy tibial plateau fractures reported in previous studies [9]. Higher complication rates, including stiffness, delayed union, and non-union, have been documented, particularly in patients with severe soft-tissue injury [10]. Functional assessment using Rasmussen’s functional scoring system demonstrated a mean score of 26.37 ± 3.3, with excellent-to-good outcomes in approximately 83% of patients, indicating acceptable short-term functional recovery in this complex injury cohort [11]. While the proportion of excellent outcomes was limited, this likely reflects the severity of fracture patterns and the inclusion of open injuries.

Radiological evaluation revealed excellent-to-good outcomes in 86.67% of patients, indicating satisfactory restoration of alignment and joint congruity in most cases. However, the presence of fair and poor outcomes in a small subset highlights the technical challenges inherent in managing comminuted periarticular fractures and underscores the influence of fracture complexity and soft-tissue status on final radiological results.

The complication profile observed in this study was manageable. Pin-tract infection, occurring in 16.67% of patients, was the most common complication and remains a recognized limitation of external fixation techniques [11]. Factors that may have contributed to pin-tract infection in the present cohort include a compromised soft-tissue envelope, local edema, suboptimal compliance with pin-site care, and prolonged external fixator retention [11]. Importantly, all infections were superficial and resolved with conservative management. Other complications, including joint stiffness, malunion, non-union, and knee instability, were infrequent, and 63.33% of patients experienced no complications, supporting the procedural safety of hybrid fixation when appropriately applied.

Clinically, hybrid external fixation represents a feasible option in selected patients with complex proximal tibial fractures, particularly when extensive internal fixation may increase the risk of soft-tissue complications [12]. Nevertheless, the findings of this study should be interpreted in light of its small sample size, single-center design, descriptive methodology, and short follow-up, which limit generalizability and preclude assessment of long-term outcomes. Larger comparative studies with extended follow-up are required to better define the role of hybrid external fixation in this challenging fracture subset [13].

Limitations

Several limitations of this study should be acknowledged. The small sample size and single-center design limit the generalizability of the findings. The descriptive, non-comparative methodology precludes direct comparison with alternative fixation techniques and does not allow causal inferences to be drawn. The relatively short follow-up duration restricts assessment of long-term outcomes, including post-traumatic osteoarthritis and implant-related complications. In addition, the absence of patient-reported outcome measures limits the evaluation of patient-perceived functional recovery and quality of life. These limitations should be considered when interpreting the results. The relatively small sample size and single-center design may limit generalizability. The non-comparative nature of the study precludes conclusions regarding superiority or equivalence with other fixation modalities. Additionally, patient-reported outcome measures were not included, which may limit comprehensive functional assessment. The absence of blinding and the lack of formal interobserver variability assessment may introduce measurement bias. Furthermore, the short follow-up duration restricts evaluation of long-term outcomes such as post-traumatic osteoarthritis.

## Conclusions

In this prospective observational study, hybrid external fixation demonstrated feasible application and acceptable short-term functional and radiological outcomes in patients with complex proximal tibial fractures, particularly in the setting of high-energy trauma and soft-tissue compromise. The procedure was associated with a manageable complication profile in the majority of patients. However, given the small sample size, descriptive study design, and short follow-up, the findings should be interpreted with caution. Larger comparative studies with longer follow-up and inclusion of patient-reported outcomes are warranted to better define the role of hybrid external fixation in the management of complex proximal tibial fractures.

## Appendices

Appendix 1

**Table 5:** Side involved (n = 30)

Side involved	Number (n)	Percentage (%)
Right	17	56.67
Left	13	43.33
Total	30	100

**Table 6:** Time interval between injury and surgery (n = 30)

Time interval	Number (n)	Percentage (%)
<24 hours	7	23.33
1-3 days	15	50
> 3 days	8	26.67
Total	30	100
Mean	2.7	
SD	1.1	

**Table 7:** Duration of surgery (mins)

Duration of surgery (mins)	Number
Mean	93.1
SD	14.18

**Table 8:** Hospital stay (days)

Hospital Stay (days)	Number
Mean	7.4
SD	1.3

**Table 9:** Postoperative weight bearing (weeks)

Postoperative weight bearing (weeks)	Number
Mean	8.68
SD	1.26
